# Simulation models for learning local skin flap design and execution: A systematic review of the literature

**DOI:** 10.3389/fsurg.2022.918912

**Published:** 2022-07-20

**Authors:** Eleni Hadjikyriacou, Thomas Goldsmith, Frances I. Bowerman, Thomas D. Dobbs, Iain S. Whitaker

**Affiliations:** ^1^ Welsh Centre for Burns and Plastic Surgery, Morriston Hospital, Swansea, United Kingdom; ^2^ Reconstructive Surgery & Regenerative Medicine Research Group, Swansea University Medical School, Swansea, United Kingdom

**Keywords:** plastic surgery training, teaching, local flaps, simulation models, local flap design, training

## Abstract

**Introduction:**

Early exposure to practical skills in surgical training is essential in order to master technically demanding procedures such as the design and execution of local skin flaps. Changes in working patterns, increasing subspecializations, centralization of surgical services, and the publication of surgeon-specific outcomes have all made hands-on-training in a clinical environment increasingly difficult to achieve for the junior surgeon. This has been further compounded by the COVID-19 pandemic. This necessitates alternative methods of surgical skills training. To date, there are no standardized or ideal simulation models for local skin flap teaching.

**Aim:**

This systematic review aims to summarize and evaluate local skin flap simulation and teaching models published in the literature.

**Materials and Methods:**

A systematic review protocol was developed and undertaken in accordance with PRISMA guidelines. Key search terms encompassed both “local skin flaps” and “models” or “surgical simulation”. These were combined using Boolean logic and used to search Embase, Medline, and the Cochrane Library. Studies were collected and screened according to the inclusion criteria. The final included articles were graded for their level of evidence and recommendation based on a modified educational Oxford Center for evidence-based medicine classification system and assessed according to the CRe-DEPTH tool for articles describing training interventions in healthcare professionals.

**Results:**

A total of 549 articles were identified, resulting in the inclusion of 16 full-text papers. Four articles used 3D simulators for local flap teaching and training, while two articles described computer simulation as an alternative method for local flap practicing. Four models were silicone based, while gelatin, Allevyn dressings, foam rubber, and ethylene-vinyl acetate-based local flap simulators were also described. Animal models such as pigs head, porcine skin, chicken leg, and rat, as well as a training model based on fresh human skin excised from body-contouring procedures, were described. Each simulation and teaching method was assessed by a group of candidates *via* a questionnaire or evaluation survey grading system. Most of the studies were graded as level of evidence 3 or 4.

**Conclusion:**

Many methods of simulation for the design and execution of local skin flaps have been described. However, most of these have been assessed only in small cohort numbers, and, therefore, larger candidate sizes and a standardized method for assessment are required. Moreover, some proposed simulators, although promising, are in a very preliminary stage of development. Further development and evaluation of promising high-fidelity models is required in order to improve training in such a complex area of surgery.

## Introduction

Surgical training has become increasingly challenging due to restricted working hours, increasing subspecializations, centralization of surgical services, and the publication of surgeon-specific outcomes (1). All these factors have contributed to limitations in practical surgical training, which have been further confounded by the COVID-19 pandemic. This has encouraged the use of simulated and model-based surgical training and education (2). Simulation training in modern teaching and surgical education allows trainees to practice procedures effectively and safely. It can also have a positive impact on operative outcomes and can provide skills easily transferrable to the clinical setting (2–4).

Local flaps are extensively utilized in soft tissue reconstruction (2), providing wound closure when direct closure is not possible through the mobilization of adjacent skin and subcutaneous tissue (2, 5). The design and execution of flaps is a highly demanding procedure with cognitive and technical difficulties, requiring the design of appropriate flaps with respect for the local anatomy to avoid distortion (6, 7). To gain confidence and expertise in such procedures, extensive exposure and practice is required, which junior trainees lack. The expectations of reaching the level of competence required in the design and execution of a variety of flaps cannot be easily achieved due to the aforementioned causes. This necessitates a realistic simulation model that could provide surgical trainees with exposure to and familiarity with both the cognitive process of planning the flap and the procedural skills of tissue mobilization. Models have the potential benefits of affording frequent practice, skill refinement, and confidence in a safe environment so that the technically challenging execution and design of local flaps can be easily achieved (8).

A flap training model has some essential prerequisites such as cost-effectiveness, multiuse, being widely accessible and available, and last but not least, to mimic tissues closely (4, 9). Many simulator models have been introduced and suggested in the literature; however, there is no standardized or ideal model that has been widely introduced for local flap teaching. The scope of our systematic review is to highlight all the available local flap simulators and teaching models. The aim is to provide a comprehensive summary of the available flap simulation methods for surgical trainees and to provide an insight into further advancements and developments for the design of an ideal surgical flap simulator.

## Methods

### Search strategy

**Figure 1:**
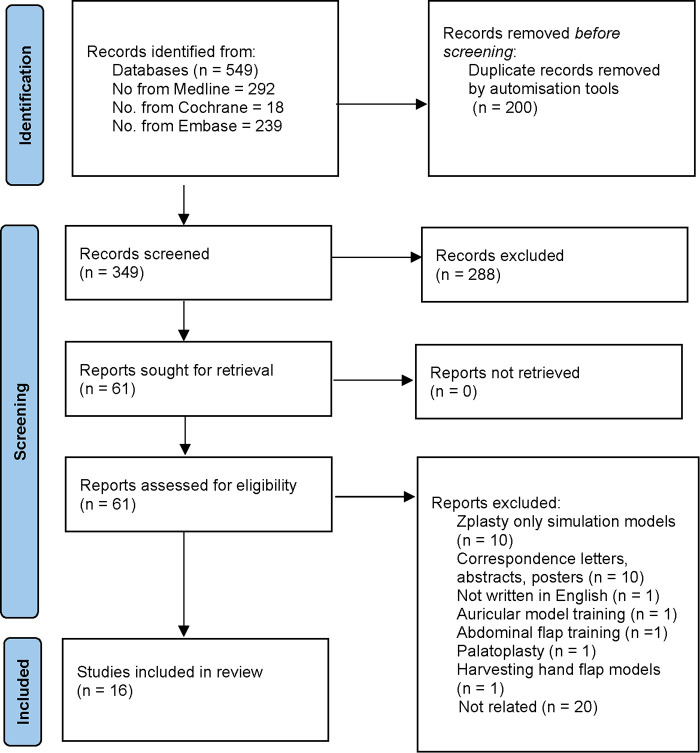
Preferred reporting items for conducting systematic reviews of included studies.

A systematic review protocol was developed in accordance with the Prisma Guidelines (Figure 1) (10). To identify all relevant papers, a comprehensive search strategy was developed. Key search terms encompassed both “local skin flaps” and “models” or “surgical simulation”. These were combined using Boolean logic and used to search Embase, Medline, and the Cochrane Library. These papers were then screened further using specific eligibility and exclusion criteria.

Inclusion criteria were all studies and articles describing teaching or simulation models for training any kind of local flap or flaps.

In addition to having a robust and reproducible search strategy, quality control was maintained by excluding any publications published as only abstracts, letters, and those not written in the English language. Furthermore, models were not developed specifically for local flap simulation, such as those for palatoplasty and abdominal flap, the auricular model and the harvesting hand flap model. In recognition of the aim of this paper to appraise models that give both planning and execution experience, models based on z-plasties alone were not included, as z-plasties, by definition, are not used to fill defects, but they redefine a scar.

### Study selection

Two reviewers (EH, FB) evaluated the studies independently with a third reviewer (TG) resolving any conflicts. The article titles were initially screened to exclude duplicates. Subsequently, the abstracts’ articles were screened using the inclusion and exclusion criteria in order to retrieve the final articles for full-text review and assessment of eligibility.

### Data extraction

Data from selected studies were extracted using Microsoft Excel 2019. The data collection included study design, type of flap procedure taught, simulation model, advantages, disadvantages, method of assessment of simulation method/training, number of candidates, origin, level of evidence, and level of recommendation.

### Data analysis

The selected studies were graded for their level of evidence and recommendation based on a modified educational Oxford Center for evidence-based medicine classification system, where the level of recommendation of 1 is the highest and 4 is the lowest (11), and assessed further according to the CRe-DEPTH tool for articles describing training interventions in healthcare professionals (12). The CRe-DEPTH tool consists of a set of reporting criteria tools for the development and evaluation of any training interventions for healthcare professionals. It consists of 12 items on 4 main domains/categories. These are (1) development of the training, (2) characteristics of the training, (3) characteristics of the providers, and (4) assessment of the training outcomes. A detailed description of each item is out of scope of this review; however, these are summarized in Table 2. The articles were separated into four different categories according to simulation model type or teaching method as described in each article as follows: (1) 3D simulation model, (2) computer and mobile app simulation models, (3) silicone-based models, (4) animal models, and (5) other material-based models such as gelatin, human skin, allevyn dressings, foam rubber, and acetic ethylene-vinyl acetate.

**Table 1:** Description of included studies with level of evidence and recommendation.

References	Origin	Study design	Type of flap procedure	Simulation model	Evaluation method	Costs	Advantages	Disadvantages – study limitations	No of candidates	Evidence	Recommendation
Yang et al.	USA	Cohort	Rhomboid flap O-T flap	3D Simulator	1. Evaluation survey – Likert scale survey 2. Rating surgical skill by a blinded experienced facial plastic and reconstructive surgeon	Not mentioned	1. Simulation as realistic training tool 2. Preferred model for assessment of trainee competency 3. Educational tool to allow for continued training during COVID-19 and scenarios requiring reduction in traditional healthcare operations	1. Small sample size 2. Confinement to a single institution and specialty	15 ENT residents	2b	3
Ederer et al.	USA	Not mentioned	Transposition flap Rotation flap Advancement flap z-plasty rotation flap	Human skin taken from patients who underwent post-bariatric body-contouring surgery	Questionnaire survey, OSATS scoring evaluation	Cost-effective	1. Realism of anatomical structure and skin elasticity allows a precise differentiation of the skin layers 2. Could be deep frozen and preserved for later use but still providing the same training qualities as fresh human skin	1. No respect to the anatomical landmarks for a location-based approach of flap-specific training 2. Small sample size 3. No correlation between the level of residency among the candidates	9	3	4
Powell et al.	USA	Not mentioned	8 types of facial flaps	Computer-aided 3D simulator	Questionnaire survey – Linkert scale survey	4.61–8.14$	1. Realistic and anatomical accuracy 2. Great potential for learning local facial flaps 3. Reusability 4. Ability to practice on the anatomical landmarks of facial features	Requires a thinner skin layer, increased elasticity, and a softer adipose layer	7 facial plastic fellowship trained ENT surgeons	3	4
Naveed et al.	England	Randomized educational trial	Not specified-local flap	Mobile simulation app – BaSSis mobile app	OSATS scoring system, task-based assessment-rated by plastic surgeon who was blinded to the allocation of participants MCQ	Not mentioned	1. Achieving a more realistic effect of wound opening during cutting by introducing additional tension to the springs between the epidermis layers	1. Unable to ensure that participants did not read more about skin surgery and local flaps outside the course 2. Small sample size	20 students	2b	3
Kite et al.	United States	Not mentioned	Rhomboid flap bilobed rotational z-plasty bilobed nasolabial forehead flap	3D simulator	Questionnaire survey	Cost-effective	Replicates elasticity of natural skin	Not mentioned	9 plastics surgery residents from 1 to 5 years - 2 faculty attending surgeons - General surgery trained burn fellow - Plastic surgery nurse practitioner	3	4
Ueda et al.	USA	Not mentioned	Local flaps not specified	3D simulator	“Enjoyable and realistic experience” “Can understand 3D movement of the flap”	Cost 30–60$	1. Enables an understanding of 2.3D design and flap movement simulates the operation of face-like structures that have complicated 3D structures	Not mentioned	6	4	4
Taylor et al.	England	Not mentioned	z-plasty rhombic flap bilobed paramedian forehead	Gelatin skin	Questionnaire survey – Linkert scale survey	Cost-effective	1. Easy to fabricate gelatin prosthetic facial skin 2. Re-usable 3. Adequately replicates facial anatomy and techniques required for rotational advancement and transposition flaps	1. Small size of evaluation 2. Limited ability to replicate tissue mobility	10 ENT residents	3	4
Mitchell et al.	USA	Not mentioned	z-plasty variants rhomboid s-plasty rotational flap dufour-mentel mouly	Computer-based simulation	Feedback	Not mentioned	Not mentioned	1. Graphics should be used to show where secondary closure stresses in the skin are 2. Surgical action recording 3. Additional indicator graphics needed 4. Additional graphics such as instructor grease pencils and anatomical details such as blood vessels	9 residents, 4 clinical and research fellows, and 5 plastic surgery faculty members	4	4
Bauer et al.	England	Not mentioned	RFF ND FTSG SSG U flap rotational flap bilobed z-plasty	Animal model – pig head	Questionnaire – Likert scale Initial written test, 8 modules of 2 h and final Dops exam test	Not mentioned	Satisfactory model for medical student training	Selection bias as participation was by choice	19 medical 7th–10th semester	3	4
Isaacson et al.	United States	Not mentioned	Rhomboid bilobed rotation island transposition flaps (suturing and z-plasties)	Animal model – feathered fresh turkey thighs	1. Questionnaire survey 2. Comparison of porcine skin to fresh turkey thighs the galliform model preferred for suturing, rotation, and advancement flaps. Thinner and more flexible than pigs skin, resembled eyelid and geriatric facial skin. porcine seemed more realistic for z-plasty, pigs skin resembled nasal and nasolabial tissues	Cost-effective	1. Low-risk zoonotic infection 2. Dermis fat and subdermal measured similarly to facial dermal thickness 3. Enough surface area for multiple procedures 4. Permit undermining rotation and tunneling. relatively elastic like human skin	Defeathering process removes epidermis altering surface the thin mobile dermis is too easy to advance and lacks a thick layer of dermal fat so poor model of nasal or forehead reconstruction	10 ENT residents 4 physicians 2 students	3	4
Hassan et al.	United States	Not mentioned	Transposition and rotation flap rhomboid, square peg in a round hole flap forehead, glabella bilobed nasolabial hatchet Abbe-Estlander McGregor cheek flap	Animal model – porcine skin on mannequin heads to give a 3D which closely resembles a cadaveric head	Feedbacks	Not mentioned	1. Similar to human skin and mimics properties of human skin 2. Trainees found that practicing on the porcine skin gave them the chance to master the basics on flap design and implementation 3. Incorporates all the relevant anatomical features, which can be drawn on if required	1. Difficulty with the flaps around the eyes ears and mouth as these structures are difficult to replicate in the model 2. Potential Ethical considerations on Muslim trainees	N/A	4	4
Denadai et al.	United States	Randomized controlled trial	Rhomboid flap	Comparison low- and high-fidelity models with a control group: didactic material low-fidelity models = rubberized line bench model and synthetic ethylene-vinyl acetate high-fidelity models = chicken leg and pig foot	1. Recording of surgical maneuvers and independently evaluated in a blinded fashion by two experienced surgeons 2. Participants completed a questionnaire on 5-point Likert scale- 3. Global rating scale adapted for flap evaluation – to objectively evaluate flap performances in eight main areas each of which was rated 5-point scale (1 is unsatisfactory and 5 is outstanding) total score = 40	Cost-effective	1. Bench model students showed quantitative increase in rhombic flap regardless of bench model fidelity 2. Simple, portable, versatile 3. Reproducible 4. Easy to purchase 5. Permits evaluation of tasks with feedback	1. Only one skill of rhombic flap assessed 2. No further evaluation of retention of flap skill 3. Evaluation of residents and final-year medical students	60 medical students	2b	3
Sifakis et al.	Netherlands	Not mentioned	Not specified-local flap	Computer simulation	Not mentioned	Not mentioned	1. Accurate simulation 2. Allows the design of new procedures – high degree of physical accuracy 3. Virtual surgical simulation-incision tool used to specify the surface swept by the virtual scalpes and define the topological change intended by the surgeon retraction tool: grasp and manipulate the skin after or in between performing incisions suture tool: stitch parts of the geometry together once they have been placed adjacent one to another	Not mentioned	N/A	4	4
Bjellerup et al.	United States	Not mentioned	A-T plasty H-plasty U-plasty rotational flap 3 point tip suture technique	Allevyn dressing,	Evaluated on a scale 1–5 by candidate	Not mentioned	1. Unlimited keeping qualities and skin like qualities when incised extended and sutured. 2. Sutures stay tight without the help of an assistant. 3. Good understanding of skin flaps mechanics. 4. Retained simulator by the student after the course allowing personal study	Not mentioned	22 dermatology residents without experience in flaps	3	4
Altinyazar et al.	Turkey	Not mentioned	Suturing, iospy, flap techniques (rotational and z-plasty	Rats used for previous experimental studies	Evaluated on a scale 1–5 by candidate	Cost-effective	1. Easier to find, in ethnic Muslim countries 2. Easy to perform and short preparation time 3. Usable 4. Excellent for simple sutures	1. Unable to perform deep sutures 2. Statistical significance increase in scores of practical procedures	16 interns 17 first-year residents	3	4

**Table 2:** Identification of Cre-depth criteria in the included studies.

References	Simulator	Description of the aim or objectives of the training	Description of the underlying theoretical framework	Description of the developmental process	Description of target population and setting of the training	Description of the educational resources	Description of the content of the training	Description of the format	Description of the didactic methods of the training	Description of tailoring of the training	Description of the providers of the training	Description of the measured outcomes	Description of the applied assessment method, including validity and reliability
Yang et al. (2)	3D/silicone-based	x	N/a	x	x	x	x	–	–	–	–	x	x
Ederer et al. (8)	Human skin	x	x	x	x	x	x	x	x	–	–	x	x
Powell et al. (13)	3D/silicone-based	x	x	N/a	x	x	N/a	N/a	N/a	N/a	N/a	x	x
Naveed et al. (14)	Mobile app	x	x	x	x	x	x	x	x	x	–	x	x
Kite et al. (15)	3D/silicone and foam-based	x	–	x	x	x	x	N/a	–	–	–	x	x
Ueda et al. (16)	3D	x	N/a	N/a	x	x	N/a	N/a	N/a	–	–	–	–
Taylor et al. (17)	Gelatin skin	x	N/a	–	x	x	N/a	N/a	–	–	x	x	–
Mitchell et al. (18)	Computer simulation	x	X	x	x	–	–	–	–	–	–	–	–
Bauer et al. (19)	Pig head	x	–	x	x	x	x	x	x	x	x	x	x
Isaacson et al. (20)	Galliform	x	N/a	–	–	x	N/a	N/a	N/a	N/a	N/a	–	–
Hassan et al. (21)	Porcine skin	x	x	–	x	–	–	x	–	–	–	–	–
Denadai et al. (22)	Low- and high-fidelity bench models	x	x	x	x	x	x	–	x	–	–	x	x
Sifakis et al. (23)	Computer simulation	x	x	x	x	x	x	–	–	–	–	–	–
Bjellerup (24)	Allevyn dressing	x	x	–	x	x	x	N/a	x	–	–	x	–
Altinyazar et al. (25)	Rat models	x	–	x	x	x	x	x	x	–	–	x	–
Dinsmore et al. (26)	Foam rubber	x	–	x	–	x	x	x	x	–	–	x	–

## Results

The initial number of studies post-duplication removal were 349. The final articles sought for retrieval were 61, leading to a final 16 articles that fit the eligibility criteria for final review. The key characteristics of the studies were (1) Type of flap procedure, (2) Simulation model, (3) Evaluation methods, (4) Advantages and Disadvantages as described for each teaching and simulation method, and (5) Number of candidates (Table 1). The models were then categorized into Computer and Virtual Simulation, 3D Simulation Models, Animal Models, and Other Models.

**Figure 2:**
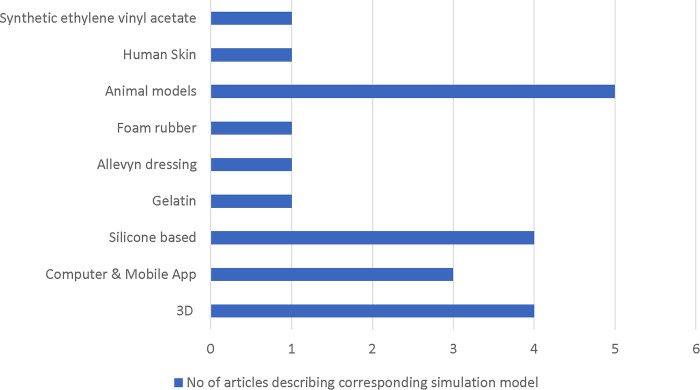
Flowchart indicating the number of included articles describing each category of simulation model.

Four articles used 3D simulators for local flap teaching and training, while two articles described computer simulation as an alternative method for local flap practicing. Four models were silicone based, while gelatin, Allevyn dressings, foam rubber, and ethylene-vinyl acetate-based local flap simulators were also described. Animal models such as pigs head, porcine skin, chicken leg and rat, as well as a training model based on fresh human skin excised from body-contouring procedures, were all described (Figure 2). Each simulation and teaching method was assessed by the group of candidates *via* a questionnaire or evaluation survey grading system. Not all studies provided the cost of production of their proposed model, making it difficult to conclude on a financial basis which was the ideal cost-effective model described so far. One cost-effective model is that of Power et al.'s, who proposed the computer-aided 3D, silicone-based model providing the cost of production estimated at 4.61–8.14$.

### Computer/virtual simulation models and mobile app

In 2009, Sifakis et al. described a virtual surgical simulation-incision tool, which is very much in its preliminary stages of development. The idea is to provide the trainee with the virtual surgical incision and retraction tool and the ability to alternate the geometry and topology of the skin and gain a better understanding on the local flap execution and design. In this system, the plastic surgeon must consider the defect created as an organic puzzle and design the optimal pattern to close the defect aesthetically and efficiently (23). Similarly, based on this model, Mitchell et al., in 2016, described a model tested on nine resident candidates. The application was able to record the user's surgical sequences. Although this has been a big advancement with great application potential, further improvements in cleft lip surgery, breast surgery, and facial flaps, such as the use of graphics to show where secondary closure stresses in the skin are the highest, surgical action recording, the need for additional indicator graphics, and the non-use of anatomical structures are required (18). Mobile simulation Apps have also been introduced by Naveed et al. with the development of algorithms and modules that aim to teach key concepts in flap execution and design. In this study, a randomized educational trial was carried out on 18 medical students, and an assessment of the application was performed with MCQs and task analysis score. The control group obtained MCQ scores and task-based assessment scores of 56.73% and 2.58, respectively, while the intervention arm had a 62.95% MCQ score and a score of 3.53 on task assessment. The task assessment score was rated from 1 to 5 and was based on multiple domains, some of which were flap planning, coverage and suturing, excision and undermining, flap marking and planning, demarcation and margins, respect of tissue, etc. This demonstrated a statistically significant difference between the intervention group and the non-intervention group (14).

### 3D simulation models

In a cohort study, Yang et al. presented a 3D-printed facial flap simulator with the aid of a CT scan, manufactured with silicone. Fifteen ENT candidates were involved in this study, with an evaluation survey on the basis of the Likert scale and a blind-folded assessment by consultants. The simulator group gave high ratings across the domains of usefulness, effectiveness, and realism of the model as a training tool. These were graded from 1 (none) to 5 (significant). The results were promising, as the overall satisfaction rate was higher. However, the sample size was small and confined to a single institute, and the mean ratings for realism, for effectiveness as a training tool, improvement in confidence levels, and expertise level were 3.22/5, 4.11/5, 3.89/5, and 3.67/5, respectively. The control group gave average to below average ratings across all survey domains. The average rating scale of 0 to 10 given by an experienced facial plastic surgeon based on the performance of both groups was 8.9 for the simulator group and 7.14 for the control group (2). Similarly, Powell et al. developed a 3-mm skin depth and a 6-mm fat depth by using CT scan. A negative casting mold was designed. Skin-colored silicone was molded on the casting mold. A ten-shore silicone was added as a second layer representing the fat layer. Seven plastic surgery and ENT trainees took a survey and evaluated the simulator on the basis of 1–4/5 Linkert scale, giving a mean domain of 3.29/4 overall on physical attributes, a mean domain of 3.19/4 on rating the realism of experience, and 4.50/5 on the performance of the flaps practiced (13). Kite et al., who studied nine plastic surgery trainees, and Ueda et al., who studied six residents, used a foam core base overlaid with fabricated multiple silicone layers to enable the layers to adhere to each other and a two-layer elastic model with the mold made by salt granules polyurethane for the surface layer and inner silicone layer, extracted by face digital imaging by using CT, MRI stereolithographic data, respectively (15, 16). In the Kite et al.’s study, 9/10 learners reported a better understanding of the local flaps theory and 8/10 candidates reported gaining more confidence in planning and execution underlying local flaps. The realistic experience of practicing undermining with the proposed flap was graded as 7/10. The flap model was scored 7/10 for simulating the design and execution of local flaps accurately (15). The candidates’ response was more generalized in Ueda et al.'s proposed model and was “an enjoyable and realistic experience” (16).

### Animal models

Our systematic review showed that pig heads could be used. However, the study contained a selection bias as candidates were selected to participate (19). Isaacson et al. described the galliform model as a low-cost and reproductive simulation model. A survey of 10 participants showed that the defeathering process removes the epidermis altering the surface, resulting in a thin mobile dermis that is too easy to advance, and lacks the thick layer of dermal fat. Therefore, this will not be adequate for nasal or forehead reconstruction (20). In these two studies, there was no candidate rating or any performed statistical analysis of the teaching method and proposed simulation model. Porcine skin on mannequin heads to give a 3D was found to exhibit similarities to cadaveric head (21). However, candidates found it challenging to practice the flaps around the eyes or mouth as these areas are difficult to replicate. This study only mentions about trainee feedback without further evaluation surveys and assessments compared with previous studies that we have seen so far. Likewise, the skin of rats used in previous experimental studies properly processed has also been described (25). Interestingly, Denadai et al. compared high- (chicken leg and pig foot) and low- (rubberized line bench model synthetic ethylene-vinyl acetate) fidelity models. This comparison showed that the high- and low-fidelity groups displayed similar post-training performances, while the groups’ confidence levels in flap performance were similar compared with that of the control group (22). Participants using the low- and high-fidelity models reported more confidence in handling the rhomboid flap post training, and compared with the control group, their confidence levels were significantly high (*P* < 0.05).

### Other models

Only a relatively small number of different techniques and methods has been described in the literature. Silicone-based models in a 3D simulator are the most described. Gelatin, allevyn, foam rubber, and synthetic ethylene-vinyl acetate are alternatives. Taylor et al. and Dinsmore et al. described gelatin- and foam rubber-based simulation models, respectively. The evaluation method in both papers relied on survey questionnaires and evaluation. However, only in the simulation model of Taylor et al., the candidates mentioned satisfaction in resemblance to fascial anatomy, with more than 80% of candidates suggesting that the gelatin model is realistic in terms of resembling the fascial anatomy and 100% opining that the model collates with the essential skills needed for fascial flap and increases the residence competency. Dinsmore et al. found that the simulator contained positive feedback relating to the basic understanding of the design, execution, biomechanics, and application of flaps (17, 26).

Most of the studies were graded as level of evidence 3 or 4 and were categorized in accordance with the Cre-Depth criteria (Tables 1 and 2). Although there are multiple variants among the proposed studies, a comparison of the level of evidence and recommendation between studies shows that the studies by Yang et al. (3D printed facial flap simulator with the aid of CT scan, manufactured with silicone), Naveed et al. (Mobile Simulation app), and Denadai et al. (high-chicken leg, pig foot-, and low-rubberized line bench model synthetic ethylene-vinyl acetate-based fidelity models) have the highest level of evidence, 2b, and the level of recommendation 3. Although measuring realism of the models is relatively objective in nature, not all studies investigated this parameter. The studies that specifically investigated the realism of the model were the silicone-based models of Yang et al., Kite et al., and Powell et al. The realistic experience was highly graded by the candidates, and, therefore, one can conclude that the 3D-based silicone models, with often some manufacturing variations, could resemble fascial anatomy. However, the data obtained in these studies are for small candidate sizes and cohort numbers, and more models are proposed models in the literature that need to be evaluated, making it difficult at this stage to flag the best simulation model.

Naveed et al.’s and Bauer et al.’s teaching method studies met most of the Cre-DePTH criteria. Naveed et al. developed novel algorithms and modules in a mobile simulation App to teach concepts required for various defect reconstruction techniques with additional resources such as videos and formal guidelines made available at relevant points in the simulation. A randomized educational trial was followed using the mobile simulation app with 18 medical students divided into intervention group learning using the new mobile simulation app, and a control group undergoing a text-based self-study. Student knowledge and skills were assessed through MCQ and task analysis. Bauer et al. perfomed two practical courses with 8 modules of 2 h for 10 students. The course modules included the surgical techniques of PRS, such as local flaps in a complex facial defect on pig heads, and were supervised by two OMFS surgeons. The identical initial and final tests examined theoretical knowledge and practical skills. Questionnaires concerning basic demographic data, future career goals, and perception of surgical disciplines before and after the completion of the course were handed out.

## Discussion

The complexity of the processes involved in the planning and execution of flap-based reconstructions is reflected by the variability in simulation models yielded by this review. The inconsistency in outcomes reported between the studies, the lack of a standardized reporting and assessment tool, as well as variation among the study designs themselves, make the drawing of any firm conclusions or assertions unfeasible. To perform a local flap-based reconstruction requires a consideration of a multitude of factors, including the availability of local donor tissue, the effects of redistributing tension on adjacent structures, and ensuring the viability of the transposed tissue. The physical steps of performing this surgery represent an extra dimension in the cognitive process required of an operating surgeon. The emphasis on which of these processes requires honing will depend on the experience of the trainee and their familiarity with dealing with the defect or flap in question. Some studies included in this review focused on the technical execution of flaps as judged by expert faculty; others reported the perceived outcomes and confidence of trainees using the models. This reflects the spectrum of skills that can be honed and addressed with simulation-based training.

Furthermore, the availability of resources will also influence the suitability of a particular flap for a specified application; where reported, the financial costs of all the models are purported to be reasonable. Clearly, these will vary and should be considered in the context of other constraints such as the availability of human or animal tissue as reported in some studies. Clearly, the financial costs associated with digital models are less straightforward to analyze, depending on whether the initial design and programming costs should be considered or whether the costs of simply accessing an established software should be considered, and will be influenced by the direct cost per user.

Several of the models reported in this review are in early stages of development. These have been included as they are doubtless of interest and significance in signposting the potential future directions of simulation training in local flap surgery.

This review is mainly limited by the quality of the included studies, and as such it is difficult to draw firm conclusions as to which model is the best.

## Conclusion

In our systematic analysis, most of the described models have been assessed only in small cohort numbers, and therefore larger candidate sizes and standardized methods for assessment are required. Moreover, some proposed simulators, although promising, are still in a very early stage of development. Further development and evaluation of promising high-fidelity models is required to improve training in a complex area of surgery such as this.

## Data Availability

The original contributions presented in the study are included in the article/Supplementary Material, further inquiries can be directed to the corresponding author/s.
